# Editorial: Multifunctional Bioactive Nanomaterials for Tissue Regeneration, Volume 2

**DOI:** 10.3389/fchem.2022.848369

**Published:** 2022-01-25

**Authors:** Bo Lei, Aldo R. Boccaccini, Xiaofeng Chen

**Affiliations:** ^1^ Frontier Institute of Science and Technology, Xi’an Jiaotong University, Xi’an, China; ^2^ Key Laboratory of Shaanxi Province for Craniofacial Precision Medicine Research, College of Stomatology, Xi’an Jiaotong University, Xi’an, China; ^3^ Department of Materials Science and Engineering, Institute of Biomaterials, University of Erlangen-Nuremberg, Erlangen, Germany; ^4^ Department of Biomedical Engineering, School of Materials Science and Engineering, South China University of Technology, Guangzhou, China

**Keywords:** bioactive, multifunctional, nanomaterials, tissue engineering, cancer therapy

The development of bioactive biomaterials is an important component in the general field of tissue engineering, specifically for the success of tissue repair and regeneration approaches (Pacelli et al., 2017). Bioactive properties including antibacterial, antiinflammatory, antitumor and antioxidant activities can efficiently regulate cell attachment, proliferation, differentiation, and the immune microenvironment, which are essential cellular functions regulating new tissue formation (Gaharwar et al., 2020). Therefore, the science and technology of bioactive biomaterials continue to be at the center of the interest of researchers in the biomedical area worldwide.

Engineered bioactive materials involve conventional biomaterials, such as bioactive glasses and ceramics, as well as biopolymers based on proteins. Polysaccharides and biomoleculecontaining biomaterials (Zheng et al., 2019). In recent years, the development of nanomaterials has brought new possibilities for tissue regeneration, due their unique surface, small size, and quantum size effects (Wang et al., 2021). Thus, nanoscale bioactive materials have attracted increasing interest in regenerative medicine recently (Chen et al., 2021; Luo et al., 2021). For example, relative to conventional bioactive glass, bioactive nanoscale glasses (BNG) have shown enhanced apatite-forming ability and osteogenic differentiation activity, as well as improved bone regeneration capability (Xue et al., 2017; Westhauser et al., 2021). Additionally, BNG also present controlled release of ions and tailorable degradation, as well as multifunctionalities, being thus interesting for a broad range of biomedical applications (Zheng et al., 2021). Up to now, BNG have demonstrated huge potential for applications in gene delivery, cancer therapy, antiinfection, immunoregulation, bioimaging, and soft tissue regeneration (Yu, et al., 2017; Niu, et al.,2021a; Niu, et al.,2021b; Rivera, et al., 2021; Sharifi, 2022). In addition to BNG, a great number of nanoscale biomaterials, both organic and inorganic nanoparticles, nanofibers and other nanostructures, are being considered for applications in tissue regeneration, usually combined with local drug and growth factor delivery.

This Research Topic is the second part on the “Multifunctional Bioactive Nanomaterials for Tissue Regeneration” series, which includes several papers demonstrating the main advances of multifunctional nanomaterials in tissue engineering. In this topic, Sprio et al. reviewed the application of biomorphic transformations to obtain nanostructured 3-D bioceramics. Yanmei Tang et al. reviewed the advances of polydopamine nanoparticles in tissue engineering applications, including the repair of bone, cartilage, skin, heart, and nerve. Fujian Zhao et al. reported tantalum-gelatin methacryloyl-bioactive glass (Ta-GelMA-BG) scaffolds which could enhance osteointegration at the early stage of implantation. Haiping Lu et al. developed Ag and MSCs-derived exosomes-contained PCL scaffold for regulating immune cells and MSCs proliferation and differentiation. Haiping Lu et al. introduced the broad application of β-TCP in tissue engineering and discussed different approaches to enhance and customize β-TCP scaffolds, including physical modification.

The editors hope that the current topic “*Multifunctional Bioactive Nanomaterials for Tissue Regeneration Part 2*” will contribute to inspire future developments of advanced bioactive nanomaterials for regenerative medicine to close the gap between research and clinical applications.
